# The Western origins of mindfulness therapy in ancient Rome

**DOI:** 10.1007/s10072-023-06651-w

**Published:** 2023-02-02

**Authors:** Andrea E. Cavanna, Giulia Purpura, Anna Riva, Renata Nacinovich, Stefano Seri

**Affiliations:** 1 Department of Neuropsychiatry, BSMHFT and University of Birmingham, Birmingham, UK; 2 Sobell Department of Motor Neuroscience and Movement Disorders, Institute of Neurology and University College London, London, UK; 3 School of Life and Health Sciences, Aston Brain Centre, Aston University, Birmingham, UK; 4 School of Medicine and Surgery, University of Milano-Bicocca, Milan, Italy; 5 Department of Neuropsychiatry, National Centre for Mental Health, 25 Vincent Drive, Birmingham, B15 2FG UK; 6 Department of Neuropsychiatry, IRCCS San Gerardo Dei Tintori, Monza, Italy

**Keywords:** Cognitive behavioural therapy, Mindfulness, Neuropsychiatry, Philosophy, Stoicism

## Abstract

Stoic philosophy has multiple parallels with cognitive behavioural therapy interventions. In their ancient texts, the Roman Stoics present a set of theoretical principles and behavioural strategies that are directly relevant to the clinical care of patients with a wide range of neuropsychiatric conditions. Mindfulness is a key component of the ‘third wave’ of modern psychotherapy that closely resembles the ancient Stoic practice of attention or ‘concentration on the present moment’. Stoic mindfulness draws attention to one of the main principles driving both Stoicism and modern psychotherapy: the assumption that cognitive activity (reasoning) mediates emotions and behaviours. This principle can be traced back to Epictetus’ Enchiridion, where he recognises that ‘men are disturbed not by things, but by the views which they take of things’. It has been shown that cognitive behavioural therapies and mindfulness-based interventions directed at patients with neuropsychiatric disorders were originally developed as Stoic-inspired treatment interventions. Both Albert Ellis and Aaron Beck (the founders of rational emotive behaviour therapy and cognitive behavioural therapy, respectively) explicitly acknowledged the role of Stoicism as the philosophical precursor of their treatment approaches. The effective implementation of evidence-based guidelines would benefit from an increased awareness of the influence of the Stoic tradition of philosophical therapy on the treatment approaches currently in use in neuropsychiatry.

## The third wave of cognitive behavioural therapy for neuropsychiatric conditions

Psychotherapy — from talking therapies to behavioural interventions — is effectively used for a multitude of psychological, behavioural, and physical symptoms and disorders: as such, it is rightfully considered as a valuable approach in both mental and physical healthcare management [1]. The link between the different forms of psychotherapy and modern-day neurological sciences is arguably twofold [2]. First, psychotherapy is the mainstay of treatment in neuropsychiatric conditions, alongside psychopharmacology and more invasive procedures. A growing body of research suggests that specific techniques such as cognitive behavioural therapy may significantly improve psychiatric symptoms that commonly occur in a range of neurological disorders [3]. In the UK, the National Institute for Health and Clinical Excellence recommends the use of cognitive behavioural therapy interventions in the treatment pathways of adult patients with depression in the context of a chronic physical health problem, with relevant implications on a wide range of neurological conditions [4]. Second, the investigation of the neural correlates of such interventions points to neurobiological models that overlap with structural and functional changes in brain regions and networks implicated in various neurological conditions [5].

Throughout the second half of the twentieth century and the beginning of the twenty-first century, three successive ‘waves’ of modern psychotherapy interventions have been recognised [6]. The first (behaviour therapy) originated from Burrhus F. Skinner’s popular doctrine of behaviourism. The transition between the first and the second wave coincides with the birth of cognitive-behavioural therapy: this passage has often been described as the clinical equivalent of the cognitive revolution that took place in the field of scientific psychology thanks to the work of Noam Chomsky and other pioneers of cognitive sciences. The revolution of the ‘second wave’ of psychotherapy consisted in expanding the previous model that postulated a direct link between environmental triggers and behavioural responses, by interposing a cognitive mediator that had been absent in the ‘first wave’ of behavioural approaches [7]. The ‘second wave’ arguably represented the clinical equivalent of the cognitive revolution that took place in the late 1950s and 1960s: its most celebrated ambassadors were Albert Ellis (1913–2007), with rational emotive behaviour therapy, and Aaron Beck (1921–2021), with cognitive therapy. Specifically, it was Albert Ellis who first acknowledged ancient philosophers as the source of the therapeutic value of rationality as cognitive mediator between environmental challenges and emotional reactions.

The ‘third wave’ of psychotherapy was heralded in a 2004 article by Steven Hayes as a group of therapies encompassing, among others, mindfulness-based cognitive therapy and acceptance and commitment therapy [6]. In 2011, the same author and his colleagues proposed ‘contextual cognitive behavioral therapy’ as a new designation for this third wave group of psychotherapy [8, 9]. Third wave cognitive behavioural therapies have become increasingly more popular over the last few years. Such therapeutic techniques recognise a range of widely used approaches, from mindfulness-focused interventions to acceptance and commitment therapy, which have been incorporated into evidence-based recommendations [10]. The use of mindfulness-based cognitive therapy and mindfulness-based stress reduction is supported by the strongest level of evidence [3]. A growing body of research suggests that mindfulness may improve neuropsychiatric symptoms that commonly occur in a range of neurological disorders [11]. Specifically, there is evidence for the effectiveness of mindfulness-based interventions in patients with movement disorders associated with behavioural symptoms, such as Parkinson disease [12], multiple sclerosis [13–16], epilepsy [17, 18], sleep disorders [19–22], headache [23–26], and chronic pain symptoms [27–29]. Patients with acquired brain pathologies such as mild traumatic brain injury [30, 31], cerebrovascular accidents [32, 33] and dementia [34–36] have also been shown to potentially benefit from mindfulness-based interventions, as well as their carers [37–39].

Mindfulness may serve as an efficacious addition to pharmacotherapy for the treatment of various psychiatric conditions [40–42]. Specifically, the role of mindfulness-based interventions is well-established in the treatment of anxiety [43, 44] and affective symptoms [45, 46]. Finally, there is preliminary evidence supporting the usefulness of mindfulness across a range of neurodevelopmental conditions, such as autism spectrum disorder [47], attention-deficit and hyperactivity disorder [48], and behavioural symptoms associated with intellectual disability [49]. Neuroimaging studies have shown an association between mindfulness-based interventions and grey matter changes, with involvement of the insula, and, possibly, the anterior cingulum and adjacent paracingulate cortex [50]. The practice of mindfulness has also been linked to reduced activity and connectivity of cortical midline structures, including the posteromedial parietal cortex (default mode network) [3]. The precuneus and adjacent posterior cingulate cortex are thought to be involved in self-referential processing, including rumination, worry, and self-criticism [51, 52]. While these findings point to a potential effect on the limbic system and default mode network, replication studies are required to reach more definitive conclusions on the neural mechanisms underlying the effectiveness of third wave cognitive behavioural therapies [53].

## Greek and Roman Stoicism as therapy

A new wave of classical studies has shed light on the role of ancient Greek philosophy as a form of psychotherapy ante litteram [54, 55]. When Hellenistic philosophies penetrated the Roman culture, they brought a set of psychological techniques aimed at restoring or preserving the healthy mind [56]. The Stoic armamentarium was found to be particularly in tune with the Roman spirit and spread throughout the Roman empire [57–59]. Stoicism owes its name to the ‘ποικίλη στοά’ (‘painted porch’), a colonnade overlooking the central square of Athens, where merchant-turned-philosopher Zeno of Citium (334–262 BCE) taught his disciples. In turn, Zeno had been exposed to the teachings of Socrates, which explains why Stoicism is remembered as a major Socratic school, alongside the rival school of Epicureanism. Crucially, the Stoic reception of the Socratic doctrines was deeply influenced by one of Socrates’ own disciples, the Cynic philosopher Antisthenes (444–365 BCE), known for his proverbial frugality. The very use of the capital S in Stoic(ism) hints at the original philosophical doctrine, as opposed to the widespread meaning of stoic(ism), which evokes the image of an emotionless person who endures pain or hardship without display of feelings. Stoic philosophy puts forward a far more nuanced approach to emotional self-regulation, which is more consistent with the aims of modern psychotherapy [60]. This misconception did not prevent Stoicism from flourishing and influencing Western culture for over two millennia.

Stoicism is traditionally divided by scholars into three phases [57–59]. The first phase, or Early Stoa, featured Zeno, the founder, and the philosophers who were the first heads of the Stoic school after him, including Cleanthes of Assos (330–230 BCE) and Chrysippus of Soli (279–206 BCE). The introduction of the Stoic teachings to Rome was achieved by two other heads of the school during the Middle Stoa, the philosophers Panaetius of Rhodes (185–109 BCE) and Posidonius of Apameia (135–51 BCE). Finally, the Late or Roman Stoa partially overlapped with the early Christian era and was characterised by the activity of Lucius Annaeus Seneca (4 BCE–65) and Musonius Rufus (25–95), the teacher of the freed slave Epictetus (50–135). By his own admission, the philosopher-emperor Marcus Aurelius (121–180) was exposed to the teachings of Epictetus, as summarised by the writings of his disciple Arrian of Nicomedia (86–160). The only surviving texts from the Early Stoa and the Middle Stoa are scattered fragments, whereas a few complete texts written by the Roman Stoics have reached us (Table 1). The Roman Stoics built on the work of their Greek predecessors, with greater focus on the practical implications of the doctrine.

**Table 1:** Chronology of main Greek and Roman Stoics, with extant works (excluding fragments/excerpts)

Philosophers	Extant works
Greek Stoics (Early and Middle Stoa)	
Zeno of Citium (334–262 BCE) — first scholarch	Nil
Cleanthes of Assos (330–230 BCE) — second scholarch	Nil
Chrysippus of Soli (279–206 BCE) — third scholarch	Nil
Zeno of Tarsus (?–180 BCE) — fourth scholarch	Nil
Diogenes of Babylon (230–142 BCE) — fifth scholarch	Nil
Antipater of Tarsus (200–129 BCE) — sixth scholarch	Nil
Panaetius of Rhodes (185–109 BCE) — seventh scholarch	Nil
Roman Stoics (Late Stoa)	
Lucius Annaeus Seneca (4 BCE–65) — politician	Essays, letters
Musonius Rufus (25–95) — teacher	Discourses*
Epictetus (50–135) — teacher	Enchiridion*, discourses*
Marcus Aurelius (121–180) — emperor	Meditations

^*^Collections of teachings recorded by students (Lucius for Musonius Rufus, Arrian of Nicomedia for Epictetus)

It could be argued that the two key elements of the Stoic doctrine are straightforward interpretations of Socrates’ teachings [57–59]. First and foremost, life ought to be conducted in accordance with nature. This naturalistic approach, which also prompted Aristotle’s investigation of nature, was epitomised in pithy ‘sententiae’. Seneca’s maxim that ‘fate leads the willing soul, but drags along the unwilling one’ (‘fata volentem ducunt, nolentem trahunt’) reverberates in the words of one of the early fathers of modern science, Francis Bacon: ‘Nature to be commanded must be obeyed’ (‘non nisi parendo natura vincitur’). Learning to accept and eventually to embrace the natural course of the events (‘amor fati’) does not necessarily lead to fatalism: the exhortation to ‘endure and abstain’ (‘sustine et abstine’, attributed in Greek to Epictetus as ‘ἀνέχου καὶ ἀπέχου’) contrasts with the proactive attitude demonstrated by the public roles of Seneca as Nero’s tutor, and of Marcus Aurelius as enlightened emperor.

The second key point the Stoic doctrine addresses is ‘ἀρετή’ or ‘virtue’ — in the ancient meaning of the word: the virtue of sharp tools like knives is about cutting, and the virtue of rational beings like us is about acting in accordance with our natural faculty of reason. It follows that the decision to adopt reason as the guide of our behaviour is the only good, whereas failing to do so is the only vice. In between, there are countless ‘ἀδιάφορα’ or ‘indifferents’: our health, our wealth, and our reputation are among them. Strictly speaking, the decision to exercise the faculty of reason is the only thing that is completely within our control. The outcomes of our actions are outside our control — despite our best intentions and efforts: accomplishments and praises belong to the realm of preferred ‘indifferents’, according to the more accommodating thinkers among the Stoics.

When did Stoic wisdom start to be seen as psychotherapy? Arguably, from the very beginning: the Socratic concept of philosophy as medicine for the soul became commonplace throughout the Hellenistic age, when the rival schools of Stoicism and Epicureanism were at least as popular as Platonism and Aristotelianism. In one of his surviving fragments, Epicurus (341–270 BCE) adopted a clinical analogy to portray the view of philosophy as therapy of the mind: ‘A philosopher’s words are empty if they do not heal the suffering of mankind. For just as medicine is useless if it does not remove sickness from the body, so philosophy is useless if it does not remove suffering from the soul’. It is therefore hardly surprising that in his discussion of the Hellenistic philosophical schools, Cicero referred to philosophy as ‘Socratic medicine’ (‘Socratica medicina’) and ‘medicine for the soul’ (‘animi medicina’). By the time Epictetus declared that ‘the philosopher’s school is […] a doctor’s clinic’, word had spread among ancient Greek historians that the inscription ‘ψυχῆς ἰατρείον’ (‘healing place of the soul’) had been carved over the entrance door of the library of Ramses II (1279–1213 BCE) at Thebes.

## The Stoic roots of cognitive behavioural therapy

A possible explanation for the early link between philosophy and psychotherapy was proposed towards the end of the twentieth century by French philosopher and classicist Pierre Hadot (1922–2010) [54]. According to Hadot, ancient discourse on logic, physics, and ethics was ultimately aimed at the practical goal of changing people’s lives. Starting from the Socratic schools, the aim of philosophy as a way of life was to ‘transform’ rather than to ‘inform’ students. Practical philosophy inaugurated a tradition of ‘spiritual gymnastics’, in Hadot’s words. Following the Renaissance and the revival of classical studies, a range of ‘spiritual exercises’ were codified in a set of religious meditations, contemplations, and prayers in the sixteenth century by Ignatius of Loyola. With the return of secularism, the role of the practical philosopher of antiquity has been revived as a guide to modern living. Specifically, analysis of the texts of the Roman Stoics suggests that different types of psychotherapy currently in use can be traced back to the Stoic tradition of philosophical therapy [61–65].

Stoicism was explicitly credited as the philosophical foundation of cognitive behavioural therapy [66, 67]. Albert Ellis, the founder of rational emotive behaviour therapy, referred to Epictetus as ‘a remarkably wise Stoic [who] pointed out some of two thousand years ago that you choose to overreact to the obnoxious behavior of others while you could more wisely choose to react in a very different manner’ [68]. Admittedly, the basis of his therapeutic approach ‘was originally discovered and stated by the ancient Stoic philosophers, especially Zeno of Citium (the founder of the school), Chrysippus (his most influential disciple), Panaetius of Rhodes (who introduced Stoicism into Rome), Cicero, Seneca, Epictetus, and Marcus Aurelius. The truths of Stoicism were perhaps best set forth by Epictetus, who, in the first century AD wrote in the Enchiridion: ‘Men are disturbed not by things, but by the views which they take of them’. Shakespeare, many centuries later, rephrased this thought in Hamlet: ‘There’s nothing good or bad but thinking makes it so’’ [69]. Despite the chronological inaccuracy (Epictetus’ words were actually written down in the ‘Εγχειρίδιον’ — literally, ‘Handbook’ — by his disciple Arrian in the first half of the second century), Ellis’ statement clearly shows that he was fully aware of his debt to his illustrious predecessor. The key difference between the ‘second wave’ of cognitive behavioural therapies and behaviourism is a crucial point: emotional disturbances and neuropsychiatric symptoms are not due to external events, but to our irrational beliefs about such events. In his first major publication on rational emotive behaviour therapy, Ellis stressed that the central premise of the emerging cognitive approach to psychotherapy could be traced back to the ancient Stoics: ‘By direct statement and implication, then, modern thinkers are tending to recognize the fact that logic and reason can, and in a sense must, play a most important role in overcoming human neurosis’. Eventually, they may be able to catch up with Epictetus in this respect, who wrote — some nineteen centuries ago — that ‘the chief concern of a wise and good man is his own reason’ [69].

**Fig. 1:**
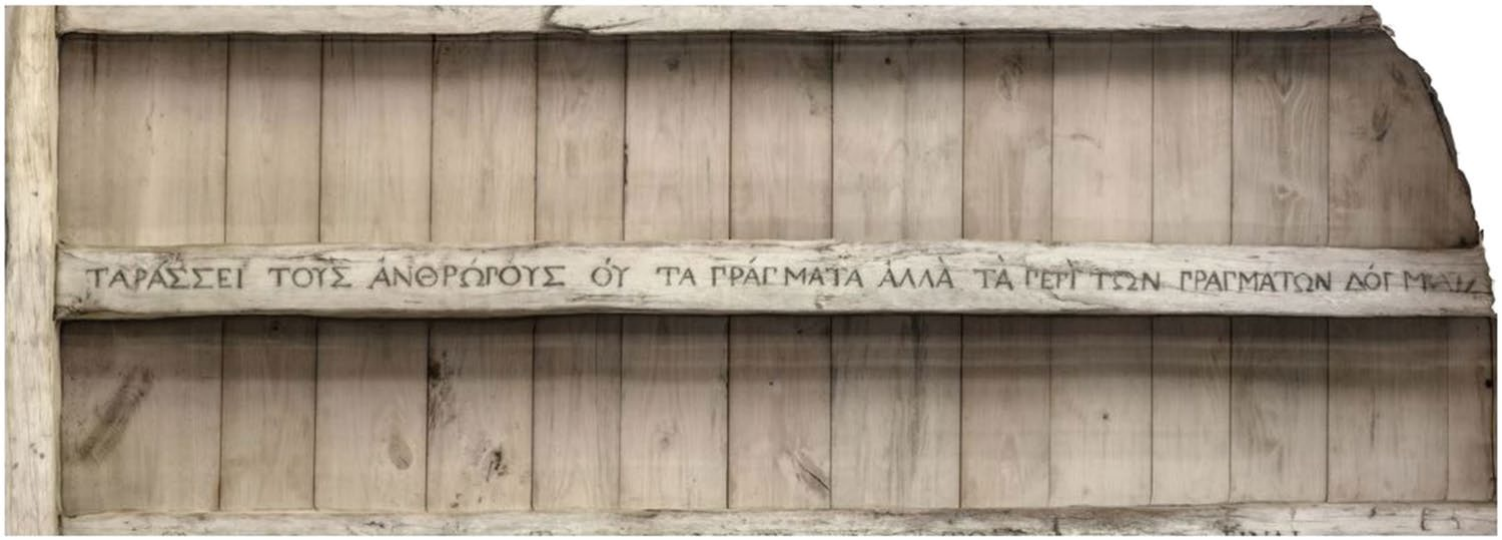
Epictetus’ words ‘ταράσσει τοὺς ἀνθρώπους οὐ τὰ πράγματα, ἀλλὰ τὰ περὶ τῶν πραγμάτων δόγματα’ (‘men are disturbed not by things, but by the views which they take of things’) in a carved beam from Michel de Montaigne’s library (Château de Montaigne, France)

A few years later, Ellis proudly claimed to have single-handedly rescued Epictetus from oblivion: ‘I am happy to say that in the 1950s I managed to bring Epictetus out of near-obscurity and make him famous all over again’ [70]. Such a bold statement might sound like an exaggeration; however, it was mainly through Ellis’ writings that Stoicism exerted its influence on Aaron Beck, the father of second-wave cognitive behavioural therapy. Beck opened his first book on cognitive therapy by acknowledging the foundations of the concept that cognitions play a central role in determining our emotions: ‘the philosophical underpinnings go back thousands of years, certainly to the time of the Stoics, who considered man’s conceptions (or misconceptions) of events rather than the events themselves as the key to his emotional upsets’ [71]. While describing his cognitive therapy approach, Beck quoted Baruch Spinoza (1631–1677) alongside the ancient Stoics: ‘I saw that all the things I feared, and which feared me had nothing good or bad in them save insofar as the mind was affected by them’ [71]. Apparently, the echoes of Epictetus’ words had not ceased to resonate in the words of some of the most influential thinkers of all times. Michel de Montaigne (1533–1592) took a somewhat more pragmatic approach, as he famously had Epictetus’ mantra among the Greek and Latin sentences carved into the beams of the rafters of his library (Fig. 1).

Of note, a contemporary of Montaigne, the humanist Justus Lipsius (1547–1606), revived ancient Stoicism by establishing Neostoicism as a widespread philosophical current of thought at the end of the Renaissance [72]. Lipsius famously justified the use of pagan philosophy (‘bonae litterae’) in place of the holy texts (‘sacrae litterae’). Based on his reading of the Roman Stoics, he developed a model of practical philosophy that is guided by reason and is immediately applicable to daily life. These ideas quickly spread and were endorsed by Guillaume Du Vair (1556–1621), the leading exponent of Neostoicism in late sixteenth century France. Long-reaching influences involved psychotherapy: between the end of the nineteenth century and the beginning of the twentieth century, the practice of a few French-speaking psychotherapists was explicitly informed by Stoicism [73]. In particular, the Swiss neurologist and psychotherapist Paul Dubois (1829–1905) founded a ‘rational persuasion’ approach to psychotherapy, which is largely forgotten today but which prefigured modern cognitive behavioural therapy in many respects [74].

However, it is with the work of English-speaking psychotherapists that Roman Stoicism was fully brought to light again as the foundation of cutting-edge treatment interventions in the Western world. In 1979, Beck and his colleagues sealed the concept that the doctrines of Stoicism constitute the philosophical origins of cognitive therapy in their groundbreaking treatment manual for clinical depression: ‘The philosophical origins of cognitive therapy can be traced back to the Stoic philosophers, particularly Zeno of Citium (fourth century BC), Chrysippus, Cicero, Seneca, Epictetus, and Marcus Aurelius. Epictetus wrote in The Enchiridion: ‘Men are disturbed not by things but by the views which they take of them’ […] Control of most intense feelings may be achieved by changing one’s ideas’ [75]. According to the most influential authors, the cognitive revolution might have taken place two thousand years before what is commonly held.

## Stoic exercises and mindfulness practice

The third wave of cognitive behavioural therapies is represented by a growing list of evidence-based treatment strategies [9]. Among these interventions, mindfulness-based therapy has gained momentum as a recommended intervention for a range of neuropsychiatric conditions [59, 60]. Mindfulness promotes self-awareness and concentration on the present moment, in order to achieve freedom from unhealthy emotions, which are rooted either in the past (e.g. depression) or in the future (e.g. anxiety). Although mindfulness has traditionally been linked to Eastern philosophies such as Buddhism, it is worth noting that its practice is not new to the Western tradition, as it can be traced back to the Stoic armamentarium of ‘spiritual exercises’ [76]. Specifically, the practice of mindfulness closely matches the Stoic exercise of attention (‘προσοχή’, which can be translated as ‘concentration on the present moment’ — or indeed ‘Stoic mindfulness’) [74, 77].

As a striking example of the Stoic form of mindfulness, in his ‘Meditations’ Marcus Aurelius reminded himself (and all of us) that those who fail to pay attention to their own thoughts and know their own minds are bound to be unfulfilled in life: ‘Through not observing what is in the mind of another a man has seldom been seen to be unhappy; but those who do not observe the movements of their own minds must of necessity be unhappy’ [55]. Marcus Aurelius’ ‘Meditations’ could be read as an early example of a modern therapy journal. As part of their cognitive behavioural treatment intervention, patients are often asked to keep a diary (journal) where they write down their thoughts and reflect on their behavioural patterns. The very practice of recording own thoughts and feelings was adopted by Marcus Aurelius in his *Meditations*, a book that was not intended for publication and is sometimes titled ‘Τὰ εἰς ἑαυτόν’ or ‘To himself’, reflecting its original purpose — an exercise of reflective practice of Stoic discipline [55].

Certain aspects of third wave approaches to cognitive behavioural therapy seem to be more in line with the Stoic conceptions of value and mindfulness, as compared to mindfulness practices derived from Buddhism, which entail greater attention to bodily states or breathing patterns [78]. Specifically, the Stoics placed considerable emphasis on the practice of focusing attention on the activity of our executive function or ‘ἡγεμονικόν’ (‘ruling faculty’). By focusing attention on the seat of our sphere of control in the present moment, it is possible to distinguish clearly between our voluntary cognition or ‘προαίρεσις’ (‘volition’ or ‘moral choice’) and our automatic thoughts or ‘φαντασίαι’ (‘involuntary impressions’). In turn, the practice of attention or Stoic mindfulness allows to take more ownership for voluntary cognition and adopting an attitude of greater detachment and indifference towards automatic thoughts, which are often the main source of distress.

The concept and practice of Stoic mindfulness sheds light on rational emotive behaviour therapy as a precursor of third-wave cognitive behavioural therapy techniques, as Ellis trained his patients to closely monitor the relationship between their thoughts, actions, and feelings, whenever they presented with distressing symptoms [78]. Such emphasis on the constant attention to one’s faculty of judgment leads to increased awareness of the distinction between voluntary thoughts/actions and external events or automatic thoughts. The Stoics described this process as the separation of our thoughts and beliefs from their objects. In addition to the separation of judgments from events, the Stoics firmly asserted this principle in their ‘dichotomy of control’. In particular, Epictetus’ Enchiridion maintains a clear distinction between what is up to us (‘τὰ ἐφ’ἡμῖν’) and what is not (‘τὰ ούκ ἐφ’ἡμῖν’). What Ellis introduced to the cognitive behavioural therapy field through the saying ‘It’s not things that upset us, but our judgements about them’ is comparable to the process called ‘cognitive distancing’ in Beck’s cognitive therapy — or ‘cognitive defusion’ in third-wave acceptance and commitment therapy. There are traces of such techniques, such as talking to thoughts as if to another person to aid defusion, in Epictetus’ own practice, as he famously instructed his Stoic students to apostrophize their distressing thoughts by saying ‘You are just an impression and not at all the thing you claim to be’ [78].

There is an interesting chronological parallelism between the renewed interest in Stoicism and the development and implementation of mindfulness-based strategies into clinical practice. The original studies published by Pierre Hadot at the end of the twentieth century focused on the role of ancient philosophy as psychotherapy [54, 55]. These influential works heralded a fruitful line of research, which culminated in an unprecedented proliferation of academic publications on Stoicism since 2007 [57–59, 79–81]. It is noteworthy that in clinical sciences there has been an exponential growth of mindfulness research since 2006, with publications mainly originating from Western countries [82], and closer attention to mindfulness-based interventions since 2010 [83]. More recently, the operationalization of Stoic principles has been proposed for a number of clinical applications, ranging from genetic counselling practice [84] to interventions for stuttering [85]. In this sense, the classics can offer valuable guides for future directions. Moreover, these observations further highlight the importance of the study of classical languages and civilizations, which can help neurology rediscover its foundations, past therapeutical approaches and even ancient pathological presentations [86, 87].

The widespread implementation of Stoicism-informed psychotherapies into evidence-based clinical guidelines should not come as a surprise. Disguised by different concepts and expressed in different languages, these techniques have never ceased to accompany the journey of Western civilisation. Their persistence over time — as well as their striking similarities with principles developed within Eastern traditions — might be considered as further evidence that they carve human nature at its joints.
